# Effect of Vascular Formed Endothelial Cell Network on the Invasive Capacity of Melanoma Using the *In Vitro* 3D Co-Culture Patterning Model

**DOI:** 10.1371/journal.pone.0103502

**Published:** 2014-07-24

**Authors:** Shuhei Yamamoto, Michael Masakuni Hotta, Mina Okochi, Hiroyuki Honda

**Affiliations:** Department of Biotechnology, Graduate School of Engineering, Nagoya University, Nagoya, Japan; Centrum Wiskunde & Informatica (CWI) & Netherlands Institute for Systems Biology, Netherlands

## Abstract

*In vitro* three dimensional (3D) cancer models were developed to observe the invasive capacity of melanoma cell spheroids co-cultured with the vascular-formed endothelial cell network. An array-like multicellular pattern of mouse melanoma cell line B16F1 was developed by magnetic cell labeling using a pin-holder device for allocation of magnetic force. When the B16F1 patterned together with a vascular network of human umbilical vein epithelial cells (HUVEC), spreading and progression were observed along the HUVEC network. The B16F1 cells over 80 µm distance from HUVEC remain in a compact spheroid shape, while B16F1 in the proximity of HUVEC aggressively changed their morphology and migrated. The mRNA expression levels of IL-6, MDR-1 and MMP-9 in B16F1 increased along with the distance the HUVEC network, and these expressions were increased by 5, 3 and 2-fold in the B16F1 close to HUVEC (within 80 µm distance) as compared to that far from HUVEC (over 80 µm distance). Our results clearly show that malignancy of tumor cells is enhanced in proximity to vascular endothelial cells and leads to intravasation.

## Introduction

Cancer invasion and metastasis are the hallmarks that transform a locally growing tumor into a systematic, metastatic, and life-threatening disease [1]. Cancer metastasis includes multiple steps: tumor cell degradation of the extracellular matrix (ECM) by a family of matrix metalloproteinases (MMPs); migration out of the primary tumor; invadion into blood vessels; adhesion of circulating tumor cells to adhesion molecules of epithelial cells in blood vessels; and degradation of the basement membrane that causes extravasation at the secondary site [1], [2]. Intercellular communication and chemotaxis play key roles in the metastatic process and can occur via direct contact and paracrine signaling between different cell types during tumor cell invasion and metastasis [3]. In particular, vascular endothelial cells that constitute the capillary and blood vessel are deeply involved in adhesion and intravasation. Subcutaneous tumorigenicity of hepatocellular carcinoma cells in nude mice was promoted by vascular endothelial cells and its invasion/metastasis associated genes were significantly up-regulated [3]. Also, since vascular endothelial cells release numerous cytokines, hormones, and growth factors such as TNF-α [4] and VEGF [5], cultured media of vascular endothelial cells including these secretory factors significantly enhanced proliferation, migration, and invasion of hepatocellular carcinoma cells *in vitro* via activation of PI3K/Akt and ERK1/2 pathways [3]. These pathways stimulate the overexpression of invasion/metastasis associated genes such as MMPs and interleukins (ILs), and these genes promote ECM degradation [6], [7], inflammation [8], angiogenesis [9], and proliferation [10]. Thus, these interactions of tumor cells with vascular endothelial cells via direct contact and paracrine signaling have been investigated.

To study the metastatic process, *in vivo* models have been developed by injection of cancer cells intravenously in mice. These experiments replicate physiological conditions [11]. However, these models are challenging for observation of all aspects of the interaction, and control of cell-cell distance and cross-talk between human cancer cells, human endothelial cells and human tissue parenchyma [12]. Traditional 2D cell culture, which is not representative of the *in vivo* environment, is thus not suitable to evaluate malignant capacity or metastasis-associated gene expression of cancer cell because it cannot mimic physiological factors that provide conditions conducive to cancer metastasis, such as ECM or intercellular interactions [13]–[15]. The *in vitro* 3D culture platforms in which cells are placed in an ECM for invasion can also provide cell spheroid formation [16] and the distribution of oxygen and metabolic products [17]–[19]; such models are difficult for visualization of intravasation events in real-time and precise control of cell-cell distance [20], [21]. Although current invasive studies using 3D microfluidic models have been developed to overcome these limitations [5], [22], [23], such studies are largely limited in single cell manipulation and the subsequent analysis of the target cell such as PCR in the closed chamber. Thus, biomimetic cell culture systems that can control cell-cell distance and evaluate the accurate progression of cancer cells in cell-to-cell and cell-to-ECM interaction are necessary for analysis of genotypic and phenotypic changes.

In response, the cellular micropatterning method can provide useful model systems to investigate intercellular interaction under a combination of multiple controllable biochemical and biophysical microenvironments, coupled with high-resolution real time imaging. Seeking to provide an effective, organized, and practical technique, we have developed a methodology for cell patterning in 3D using magnetic force and magnetite nanoparticles [24]–[28]. Magnetite nanoparticles embedded in cationic liposomes are used for labeling cells via electrostatic interactions between magnetic cationic liposomes (MCLs) and the target cell membrane [29]. Magnetically labelled cells can then be arranged for observation. Labeling cells with MCL has little effect on cell viability, growth, and differentiation [26]. Utilizing a pin-holder device, the magnetic field of a neodymium magnet is concentrated at the peak of each pin, thus allocating a specific number of cells in a planar fashion according to seeding density, at each point of ECM (collagen or Matrigel). The arrangement of pins, cell seeding density, and cell types can be designed for the evaluation of various cell-cell interactions, and succeed in analyzing the invasion capacity of cancer cells [27], [28]. We have also succeeded in forming a vascular network of magnetically labeled HUVEC that was patterned on the Matrigel angiogenesis model [26], and in genetic analysis the effect of fibroblasts on cancer invasion in direct-interaction, indirect-interaction, and fibroblast sheet interaction models for invasion models [28]. This novel approach has the benefit of cost effectiveness, repeatability, and ease of observation for evaluation of the cell-to-cell interaction including the invasion capacity of cancer cells.

In the present study, the tumor microenvironment mimetic culture array was utilized to observe intercellular behavior of cancer cells in a 3D condition co-cultured with endothelial cells. The highly metastatic mouse melanoma cell line B16F1 was used as the cancer model, while human umbilical vein endothelial cells (HUVEC) were used as the human endothelial cell model. B16F1 cells were arranged magnetically on vascular-formed HUVEC in ECM, forming cell spheroids (cell aggregates), with a magnetic force-based pin-holder device for observation of the cancer invasion. Since this patterning method was able to control the cell-cell distance between the cancer cell spheroid and HUVEC network, our model was suitable for observation of intercellular interaction via direct contact or paracrine signaling during cancer cell invasion. In addition, gene expression of IL-6, MDR-1, and MMP-9 in the picked-up B16F1 spheroids was used to evaluate the effect of spatial relationship to endothelial cells (HUVEC) on the invasive capacity of melanoma cells. B16F1 spheroids were picked up using a manipulator and analyzed at each distance from HUVEC. Given its ability to perform cell-based assays in the tumor mimetic microenvironment in a simple and objective manner, this system may be useful in understanding the key mechanisms of invasion and drug screening for attenuating metastasis.

## Materials and Methods

### Cell culture

Mouse melanoma cells, B16F1 (ATCC CRL-6323), were grown on 10 cm dishes cultured in Dulbecco's Modified Eagle Medium high glucose (DMEM, Invitrogen, Gaithersburg, MD, USA) to which was added 10% fetal bovine serum (Invitrogen), 0.1 µg/mL streptomycin sulfate, and 100 U/mL potassium penicillin G (Invitrogen).

Human umbilical vein endothelial cells (HUVEC) were provided as frozen cells after primary culture by the supplier (Kurabo, Osaka, Japan). HUVEC were utilized as a model for human endothelial cells, and cultured on 10 cm dishes in HuMedia-EB2 (Kurabo, Osaka, Japan) consisting of 2% fetal bovine serum, 10 ng/ml human epidermal growth factor (hEGF), 1.34 µg/mL hydrocortisone hemisuccinate, 50 µg/mL Gentamicin, 50 ng/mL Amphotericin B, 5 ng/mL hEGF-B, and 10 µg/mL heparin, all supplied by Kurabo. Cells were cultured in a humidified 5% CO_2_ incubator at 37°C.

### Preparation of MCL and magnetic cell labeling

MCL was prepared as described previously [28] using magnetite nanoparticles (Fe_3_O_4_, average diameter of 10 nm, Toda Kogyo Co., Hiroshima, Japan) dispersed via sonication into colloidal magnetite nanoparticles and a lipid mixture of N-(a-trimethylammonioacetyl) didodecyl-D-glutamate chloride (TMAG), dilauroyl phosphatidylcholine (DLPC), and dioleoylphosphatidylethanolamine (DOPE) at a molar ratio of 1∶2∶2. Magnetite concentration was measured using the potassium thiocyanate method [24].

For magnetic labeling, B16F1 cells were cultured until sub-confluent. DMEM was exchanged for fresh DMEM containing finely dispersed MCL at 100 pg-magnetite per cell for 2 h incubation. Cells were then washed twice with PBS to remove non-introduced residual MCL. Magnetically labeled cells were then collected with 0.25% trypsin-EDTA (Gibco, Carlsbad, CA, USA). To evaluate the toxic effect on the cell growth of the magnetically labeling using MCL, B16F1 cells with or without magnetic labeling were grown on 10 cm dishes cultured in DMEM at seeding densities of 2×10^5^ cells/dish, and the living cell numbers were counted by trypan blue exclusion every 24 hours.

### Fabrication of pin-holder device

The pin-holder device was fabricated to allot magnetically labeled cells by the profile of their magnetic distribution [28]. Each has a base of magnetic soft iron, measuring 20 mm width ×20 mm length ×10 mm height. A wire electrical discharge machine (DIAX-FX10, Mitsubishi Electric Co., Tokyo, Japan) was used with a cutting wire (diameter of wire: 0.1 mm, Sumitomo Electric Industries, Osaka, Japan) to construct the array of pillars of square pole type, with dimensions of 100 µm width ×100 µm length ×320 µm height at intervals of 150 µm with center to center spacing of 250 µm, or intervals of 900 µm with center to center spacing of 1000 µm. Magnetic field can be concentrated on the pillars using an external neodymium disc magnet (Niruko Factories Co., Shiga, Japan; 50 mm in diameter, 10 mm in height, with surface magnetic induction 0.38 T).

### Preparation of B16F1 melanoma spheroid 3D cell culture array with HUVEC network

Collagen mixture was prepared by mixing a 7∶2∶1 volume ratio of ice-cold collagen solution, 0.3% Cellmatrix Type I-A (Nitta Gelatin, Osaka, Japan) with 5×DMEM and 10× sterile reconstitution buffer (2.2 g NaHCO_3_ in 100 mL of 0.05 M NaOH and 0.2 nM 2-[4-(2-hydroxyethyl)-1-piperazinyl]ethane sulfonic acid (HEPES)) as described in the instructions. Gas-permeable tissue culture dishes (hydrophilic lumox dish, 35 mm, SARSTEDT, Nümbrecht-Rommelsdorf, Germany) were used for the 3D culture array.

**Figure 1:**
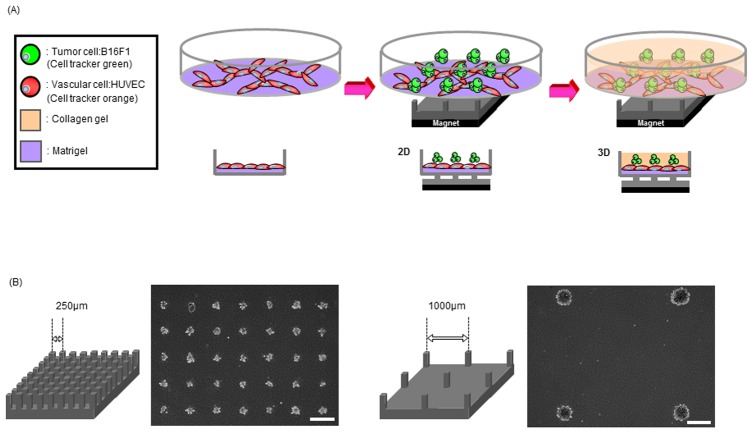
Magnetic force-based cell patterning using the pin-holder device for observation of invasive capacity of B16F1 melanoma associated with HUVEC. (A) Schematic diagram for fabrication of the 3D cell culture array. HUVEC was inoculated on a thin layer of Matrigel for network formation. The cell culture dish was placed on the pin-holder device which is placed on the neodymium magnet. The B16F1, magnetically labeled with MCL, were patterned by magnetic force. The patterned cells were then embedded with collagen gel. (B) Phase microscopic images of magnetically patterned B16F1 melanoma cells using the pin-holder device with different spacing. The center-to-center distance of the pin-holder device was 250 µm (left) or 1000 µm (right) and the cells were arranged on pins according to magnetic force. Scale bars are 100 µm.

First, a thin layer of Matrigel (100 µl) (Becton, Dickinson and Company, Cedex, France) was spread on each dish with a cell scraper. Then, HUVEC stained with CellTracker orange (CMTMR; Molecular Probes, Eugene, OR, USA) were plated on each dish at seeding density of 3×10^5^ cells/dish, and incubated overnight for network formation. The B16 melanoma cells were magnetically labeled with MCL and stained with CellTracker green (CMFDA; Molecular Probes, Eugene, OR, USA) in DMEM. For cell arrangement, the neodymium disc magnet, the pin-holder device, and the gas-permeable tissue dish with networked HUVEC were arranged in order (Fig. 1), and 2.5 ml cell suspension of the B16F1 in DMEM at seeding densities of 7.2×10^4^ cells/ml (average 10 cells/spot, 250 µm interval pin-holder); 2.16×104 cells/ml (average 1.5 cells/spot, 250 µm interval pin-holder), 2.16×10^4^ cells/ml (average 50 cells/spot, 1000 µm interval pin-holder) were inoculated onto the dish, followed by 30 min incubation. Then, after removing supernatant medium, 1 ml collagen mixture was overlaid, and the dish was removed from the pin-holder device and magnet. The dish was returned to the incubator for another 30 min for solidification of collagen, followed by the addition of 1 ml DMEM.

### Cell observation

Cells in the 3D cell array were observed via time-lapse monitoring through phase microscopy (Model IX81, Olympus Co., Tokyo, Japan). Analysis of images was performed by image analysis software (MetaMorph, Universal Imaging Co., Downingtown, PA, USA) to calculate the length of B16F1 spheroids and the distance between B16F1 spheroid and networked HUVEC. The invasion of B16F1 cells to HUVEC network were analyzed using a confocal microscope (A1Rsi-N, Nikon, Tokyo, Japan) and confocal image analysis software (NIS-Elements, Nikon, Tokyo, Japan).

### Gene expression analysis

The B16F1 cells co-cultured with HUVEC and in isolation, were collected from the 3D cell array, and treated with 0.033% collagenase. Cells were washed with PBS, counted by fluorescent microscopy, and lysed using lysis enhancer and resuspension buffer from CellsDirect One-step qRT-PCR kits (Invitrogen). Real-time RT-PCR assays were conducted on an ABI StepOne Real Time PCR Systems, using SYBR Green RNA 1step kit (Applied Biosystems, Branchburg, NJ, USA). Primers were purchased from Greiner Bio One, Frickenhausen, Germany. The sequences of B16F1 specific primers for mouse mRNA are listed in Table 1. The comparative threshold cycle (Ct) method was used to quantify gene expression in each sample. Normalization of gene expression was performed using GAPDH as a reference gene, and all data was expressed as a ratio to the reference sample of B16F1 monoculture. In order to analyze the target cell expression, we collected the cells using a micromanipulator (CellTram vario 5176, Eppendorf, Humberg, Germany). One spheroid collected by a micro manipulator were suspended into a lysis buffer directly, and the expression ratio in each spheroid was analyzed as described above.

**Table 1:** Sequences of primers for RT-PCR.

	Human (sequence 5′-3′)		Mouse (sequence 5′-3′)	
	Forward	Reverse	Forward	Reverse
GAPDH	CCTGACCTGCCGTCTAGAAA	TGCTGTAGCCAAATTCGTTG	AAGGGCTCATGACCACAGTC	CACTGGGGGTAGGAACAC
IL-6	TAGCCTCAATGACGACCTAAGCT	GGGCTGATTGGAAACCTTATTAAG	GAGGATACCACTCCCAACAGACC	AAGTGCATCATCGTTGTTCATACA
MMP-9	TGGGTGTACGACGGTGAAAA	CATGGGTCTCTAGCCTGATA	GCATACTTGTACCGCTATGG	TAACCGGAGGTGCAAACTGG
MDR1	CTGGTGTTTGGAGAAATGACAG	CCCAGTGAAAAATGTTGCCATTGAC	AACACAGCCAACCTTGGAAC	TGTTGCAATCTTTCCAGCAG

## Results

### Effects of ECM mimetic gels on cell morphology

**Figure 2:**
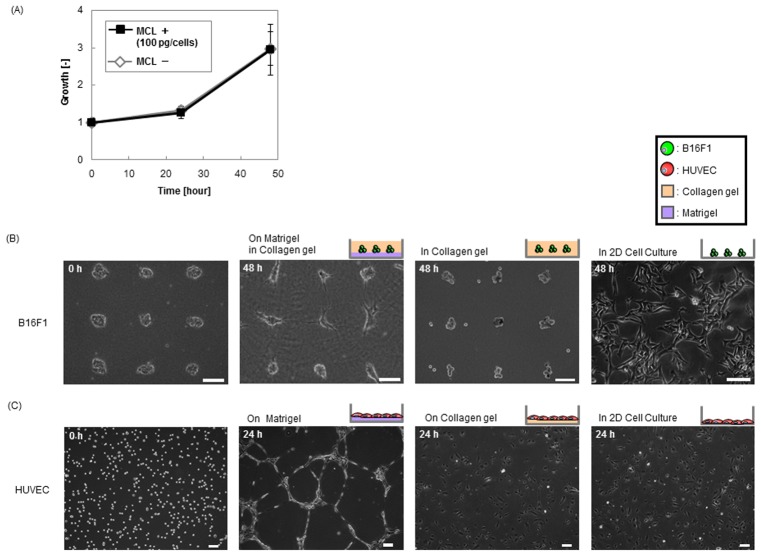
Phase microscopic images of B16F1 melanoma and HUVEC monoculture in 3D and 2D. (A) Growth curve of B16F1 with or without magnetic cell labeling by MCL in 2D cell culture. B16F1 with or without magnetic cell labeling were grown on 10 cm dishes cultured in DMEM at seeding densities of 2×10^5^ cells/dish, and the living cell numbers were counted by trypan blue exclusion at each time points. (B) Phase microscopic images of B16F1 cells. Magnetically pattered B16F1 at seeding densities of 7.2×10^4^ cells/ml (10 cells/spheroid, 250 µm interval pin-holder) with or without a layer of Matrigel was embedded with overlaid collagen (3D culture). The 2D culture was performed in comparison. (C) Phase microscopic images of HUVEC cells plated on Matrigel with or without a layer of Matrigel were embedded with overlaid collagen (3D culture) at seeding density of 3×10^5^ cells/dish. The 2D culture was performed in comparison. Scale bars are 100 µm.

To construct the *in vitro* 3D cell culture array to observe the invasive capacity of B16F1 melanoma cell spheroids co-cultured with the HUVEC network, cell morphological behaviors of HUVEC and B16F1 were investigated. Fig. 2 displays images of B16F1 and HUVEC cultured using two types of ECM-mimetic gel containing collagen type-I and Matrigel. The B16F1 were plated in 3D cellular array monoculture in collagen gel, with or without a base layer of Matrigel, as well as images from 2D culture. B16F1 cells were magnetically labeled using MCL, and then patterned in ECM mimetic gel. Since there was no significant difference regardless of MCL-labeling in B16F1 cell proliferation (Fig. 2A), MCL labeling has little effect on cell-to-cell interaction [27], [28]. After 48 h of culturing, B16F1 cells in monoculture, patterned in collagen gel at seeding densities of 7.2×10^4^ cells/ml (10 cells/spot, 250 µm interval pin-holder), formed spheroids at each locus (Fig. 2B). Although, B16F1 was highly invasive melanoma cells, the cells remained in compact spheroids, and showed no signs of invasive behavior such as elongation and invadopodia. In contrast, B16F1 showed spindle formation in the traditional 2D culture, which vastly differ from the spheroid formation *in vivo*. Thus, a more biomimetic microenvironment would be necessary to observe the invasive characteristics of malignant melanoma. When B16F1 cells were patterned in collagen gel with a Matrigel base layer, B16F1 formed multicellular spheroids with satellites of invadopodia. Invadopodia contain many type of proteases, stress fibers and adhesion proteins, indicating that their main function is to provide traction for invasive cancer cells [30]. Also, a Matrigel base layer induced the vascular-like networks of HUVEC which were plated at seeding density of 3×10^5^ cells/dish, morphology indicative of angiogenesis. However, HUVEC showed spindle formation on a collagen base layer and in 2D culture (Fig.2C). It was confirmed that Matrigel provides several angiogenesis factors such as VEGF or bFGF [31]. Hence, micropatterning in a collagen gel layer with Matrigel was suitable to observe invasive associated interaction of malignant melanoma cells with vascular endothelial cells.

### Melanoma cell behavior in 3D co-culture array with HUVEC

**Figure 3:**
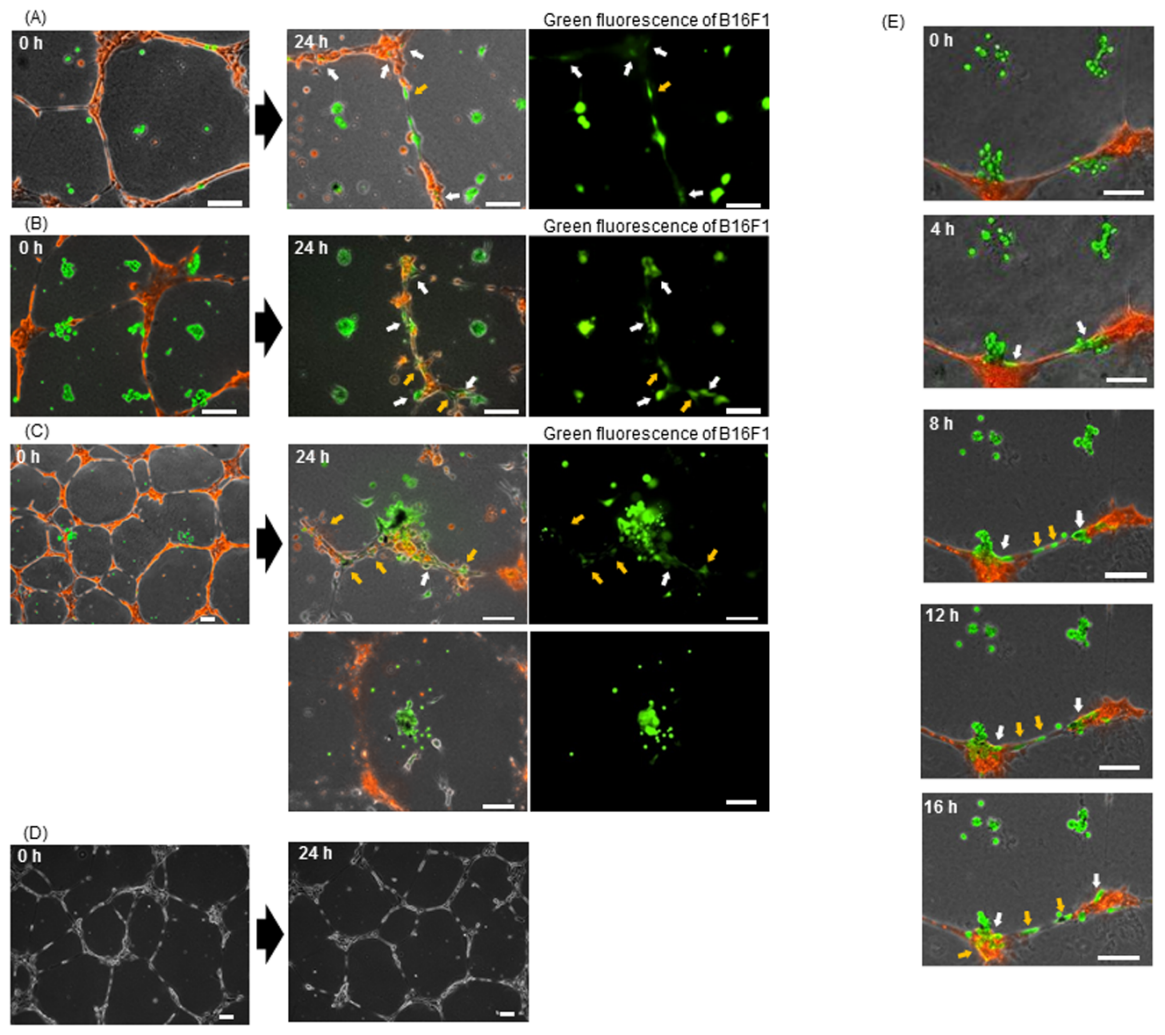
Fluorescent microscopic images of B16F1 (green) in 3D cell culture array with HUVEC network (red). Magnetically labeled B16F1 cells were arrayed at varying seed-densities over HUVEC network: 2.16×10^4^ cells/ml (A: average 1.5 cells/spheroid, 250 µm interval pin-holder); 7.2×10^4^ cells/ml (B: average 10 cells/spheroid, 250 µm interval pin-holder) 2.16×10^4^ cells/ml (C: average 50 cells/spheroid, 1000 µm interval pin-holder); and 0 cells/ml (D). Time-lapse images were taken for three plates on 0 h and after 24 h (A–D). (E) The co-culture array was fabricated at B16F1 seeding density of average 10 cells/spot, and time-lapse images were obtained at 4 h intervals from 0 h to 16 h. White arrows highlight B16F1 cells that have invaded the HUVEC network. Yellow arrows indicate the B16F1 that have spread along to the HUVEC network. Scale bar: 100 µm.

Fig. 3 shows the effect of the HUVEC network on morphological behaviors of melanoma in a biomimetic 3D co-culture array that was constructed on a Matrigel base layer in collagen type-I gel. HUVEC were plated on tissue culture dishes coated with Matrigel and allowed to network at 24 h, and then magnetically labeled B16F1 cells were arrayed over-top in networked HUVEC and set in collagen gel. We also have adjusted the patterning interval and the seeding B16F1 cell number in a spheroid for evaluation of the invasive capacity. Fig. 3A–3E, illustrate time-lapse images at varying B16F1 seeding densities and patterning interval: average 1.5 cells/spheroid with 250 µm patterning (Fig. 3A), average 10 cells/spheroid with 250 µm intervals (Fig. 3B, 3E), average 50 cells/spheroid with 1000 µm intervals (Fig. 3C), and 0 cells/spheroid (Fig. 3D), respectively. From Fig. 3A–3C and 3E, clear invasive behaviors of B16F1, such as cell elongation along to the HUVEC network, were observed in 3D co-culture with HUVEC in every seeding density and patterning interval. It seems that B16F1 that were close to HUVEC have aggressively elongated and invaded according to the HUVEC network, while B16F1 cells distant from HUVEC remain in compact spheroids (Fig. 3A, 3B, 3E). The white arrows indicate the B16F1 cells that have not only formed invadopodia but also completely invaded the HUVEC network. The B16F1 spheroids that were close to HUVEC migrated along to the pre-existing vascular network (yellow arrows). In contrast, melanoma spheroids did not show such aggressive invasive behavior in monoculture (Fig. 2B). The intercellular junction of HUVEC network became more weak, and some networks were broken in the B16F1 invasion in co-culture array (Fig.3A–3C), compared with that in monoculture of HUVEC network (Fig.3D). The secretion factors of HUVEC had up-regulated the invasion/metastasis associated gene expressions in cancer cells, including MMP-9 [3]. It seemed that the intercellular adhesion and extracellular matrix was degraded by the cancer-produced proteases. It was shown that cancer cells close to vascular endothelial cell had invaded, and the malignancy progressed rapidly. However, since the single cancer cell spots may not show the activation of migration to the HUVEC network via cancer cell-to-cancer cell adhesion such as N-cadherin signaling [32], the single cell patterning would be not suitable to evaluate the aggressive invasion of cancer cell. Also, since two morphological changes within a spheroid, such as dispersion from the spheroid or cell elongation, was observed in the big melanoma spheroids of 50 cells, (Fig. 3C; middle), it was difficult to characterize the factors relating to such melanoma invasive capacity (direct contact or paracrine signaling). We adopted 10 cells for the cell number in a spheroid to observe invasive behavior. In addition, since the average spacing between strands in the HUVEC network was 350 µm in Fig. 2C, we adopted a 250 µm patterning interval, which can set variation in distance from HUVEC for co-culture array patterning.

**Figure 4:**
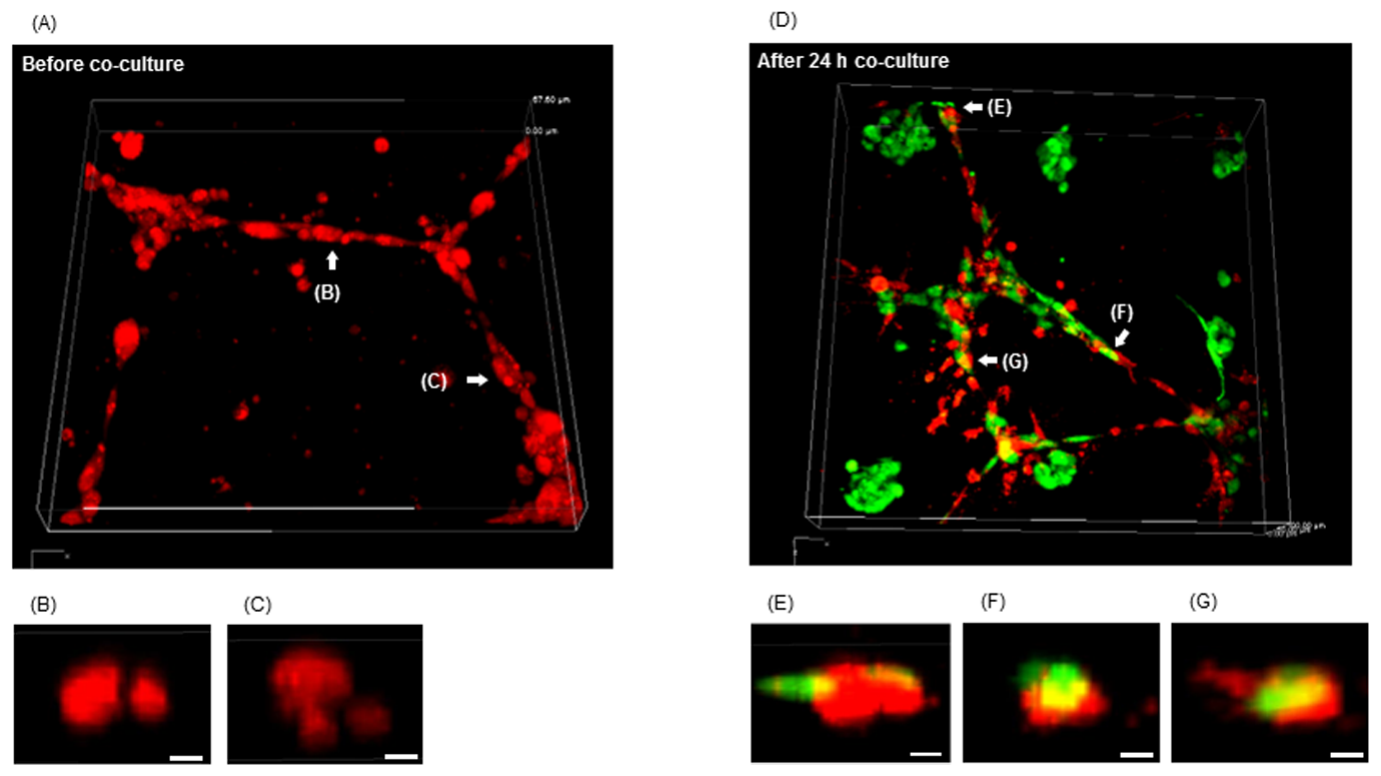
Confocal microscopic images of B16F1 (green) invasion of HUVEC network (red). Magnetically labeled B16F1 cells were arrayed at 10 cells/spheroid with 250 µm interval over the HUVEC network, and images surrounding the HUVEC network were taken before (A–C) and after the 24 h co-culture (D–G). The representative sectional view of the HUVEC network before co-culture with B16F1 (B, C) and invasive points of B16F1 after a 24 h culture (E–G). Scale bar: 10 µm.

To investigate the invasion of B16F1 cells to the HUVEC network, confocal image analysis of B16F1 cells surrounding the HUVEC network was obtained (Fig. 4). Fig. 4 shows the confocal images of HUVEC network before (Fig. 4A–C) and after 24 h co-culture with B16F1 array at 10 cells/spheroid with 250 µm interval (Fig. 4D–G). In the sectional view, network formation of HUVEC was observed (Fig. 4B, 4C). In contrast, invasion of B16F1 cells along to the HUVEC network was observed after co-culture (Fig. 4E–G). It was demonstrated that B16F1 that were close to HUVEC have invaded the endothelial network, while B16F1 cells distant from HUVEC remain in compact spheroids.

**Figure 5:**
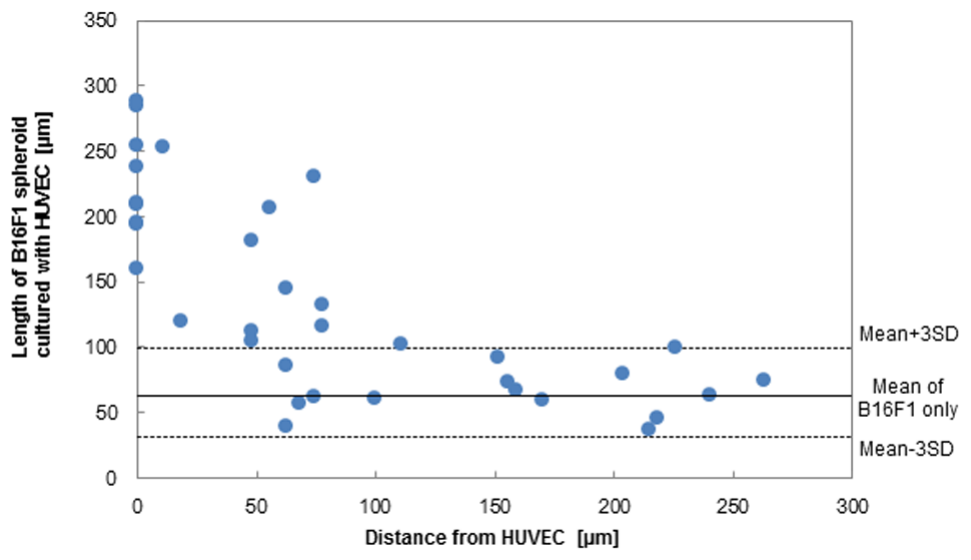
The length of B16F1 spheroids co-cultured with HUVEC network. The length of B16F1 cell spheroids patterned in 10 cells/spheroid with 250 µm interval were image-analyzed by the green fluorescence after a 24 h culture with the HUVEC network. The plot represents the length of each B16F1 spheroid. The solid and dotted lines show the average length and the average length ±3× SD of B16F1 cell spheroids in 3D cell monoculture array.

To evaluate these invasive elongations of cancer spheroids, the length of cancer cell spheroids was analyzed. Since the elongated cancer spheroid reflects the activation of invasiveness or the high invasive capacity of the cancer cells, we calculated the length of a cancer spheroid from the time-lapse image to quantify the invasive capacity of the cancer cell. Fig. 5 shows the length of B16F1 spheroids in each distance from the co-cultured HUVEC network, in which B16F1 cells were set to 10 cells/spheroid at 250 µm interval. A plot of distance 0 in Fig. 5 represents a length of B16F1 spheroid adhered to HUVEC directly, and the others, which did not adhere to HUVEC. After culturing for 24 h, B16F1 spheroids were elongated in the proximity of the HUVEC network in co-culture conditions, and the length was clearly increased within an 80 µm distance from HUVEC regardless of B16F1-to-HUVEC adhesion, while the length of B16F1 spheroids was slightly changed in monoculture. On the other hand, the length of B16F1 80 µm far from HUVEC did not significantly differ from the monoculture. Therefore, it was shown that a vascular network of endothelial cells crucially affected invasive behavior of cancer cells according to the distance to cancer cells by the secreted soluble factors, as well as by direct interaction.

### Gene expression of melanoma cells in 3D co-culture array with HUVEC

**Figure 6:**
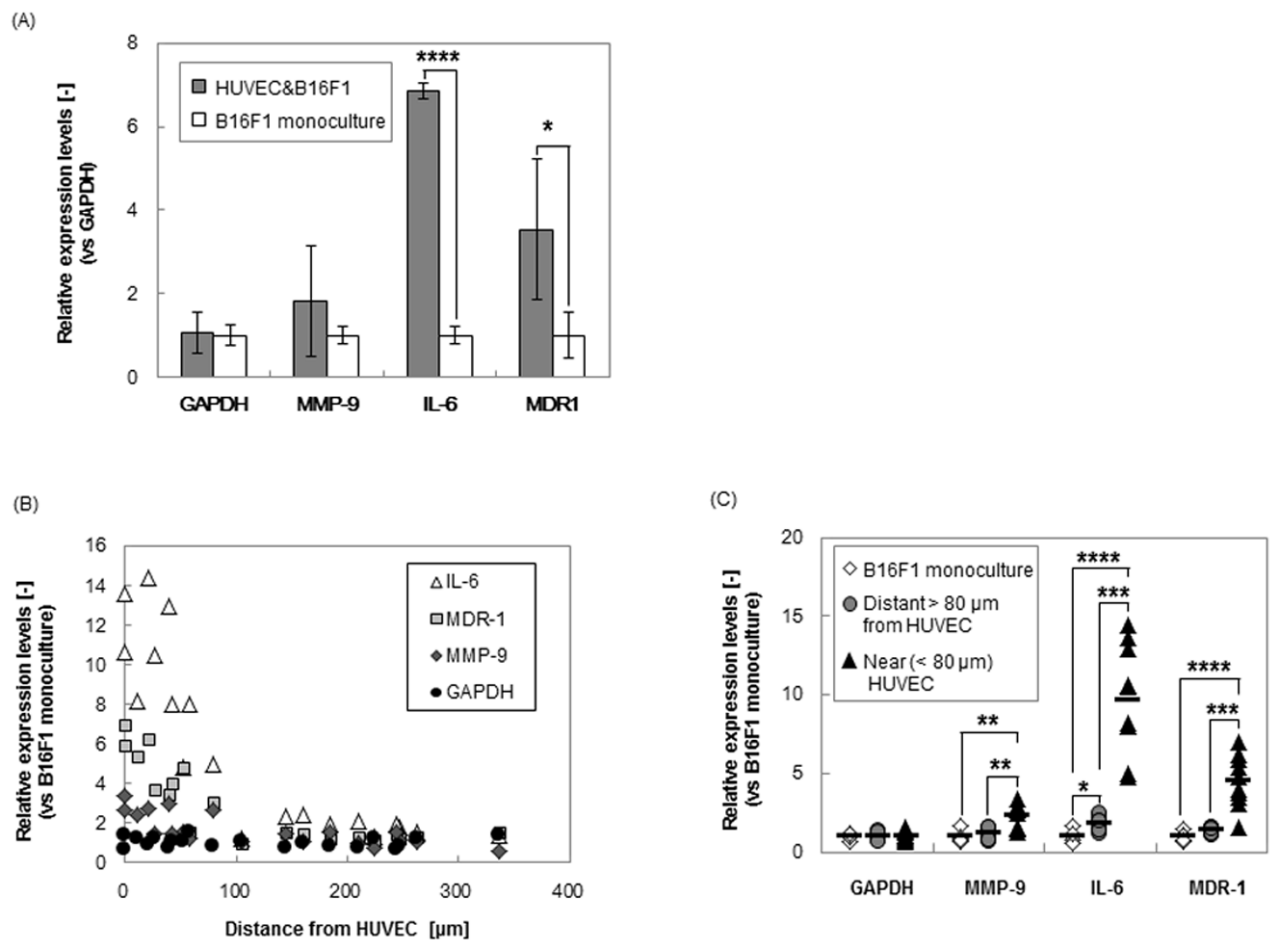
Gene expression of MMP-9, IL-6, and MDR-1 in B16F1 spheroids co-cultured with HUVEC network after 1-day culture. (A)Relative expression levels of MMP-9, IL-6, and MDR-1 in B16F1 cells of a whole culture dish. The asterisks indicated that P value was regarded as a significant difference compared to B16F1 monoculture group (*p<0.05, ****p<5×10^−5^). Data points represent means ± SD of 3 independent experiments. (B)Relative expression levels in each spheroid were plotted with the distance from the nearest HUVEC network. Each spheroid was picked-up using a micromanipulator. (C)The expression levels in each B16F1spheroid placed near (≤80 µm) and distant (>80 µm) to HUVEC was compared. Expression data was normalized to each gene expressions found in B16F1 monoculture using GAPDH as the reference gene. The asterisks indicated that P value was regarded as a significant difference (*p<0.05, **p<0.005, ***p<1×10^−4^, ****p<5×10^−5^, n = 5–10).

To observe the effect on tumor associated gene expression of B16F1 melanoma (IL-6, MMP-9 and MDR-1) patterned on the HUVEC network, real-time PCR analysis was investigated. IL-6 and MMP-9 promote invasion and metastasis of cancer cells via accelerating ECM degradation, inflammation, angiogenesis and proliferation [6]–[10]. MDR-1 is a gene that leads to the production of ATP-driven efflux transporter Pgp-170, the most common cause of multidrug resistance in many types of solid and hematological human cancers [33]. It is known that these gene expressions are stimulated by paracrine signaling such as TNF-α [4] or direct contact [34]. Fig. 6 shows that the relative expressions of IL-6, MMP-9 and MDR-1 in co-cultured B16F1 with HUVEC compared to that in B16F1 monoculture.

First, we compared the average expression of whole B16F1 cells co-cultured with HUVEC in a culture dish to that without HUVEC (Fig. 6A). To collect whole B16F1 cells, B16F1 were treated with collagenase after a 24 h culture with or without HUVEC. The mRNA expressions of collected B16F1 cells were analyzed by real time RT-PCR using specific primers for mouse mRNA to detect the B16F1 expression without any separation steps of B16F1 from co-existing HUVEC, and glyceraldehyde-3-phosphate-dehydrogenase (GAPDH) mRNA level was used as the reference. GAPDH levels in co-culture and in monoculture were relatively identical, and MMP-9 levels also did not significantly differ from B16F1 monoculture. In contrast, when B16F1 co-cultured with HUVEC, IL-6 and MDR-1 production was 7-fold and 4-fold higher, respectively. It was shown that the intracellular interaction with vascular endothelial cells was important for the progression of malignancy in cancer cells.

Next, since the invasive elongation of B16F1 spheroids was increased along to the distance from HUVEC (Figs. 3, 4, 5), we picked up the B16F1 cell spheroids using a micromanipulator, and compared the gene expressions (Fig. 6B, 6C). We could collect 6–10 cells from a B16F1 spheroid, respectively, and analyzed the mRNA expressions of all collected cells. The distance between B16F1 and HUVEC was calculated from the fluorescence images after 24 h co-culturing. Fig. 6B and 6C showed that the relative expression levels of a co-cultured B16F1 spheroid in each distance from HUVEC, compared to that of B16F1 monoculture. The mRNA expression levels of IL-6, MDR-1 and MMP-9 in B16F1 increased in inverse proportion to the distance of the HUVEC network, and these expressions were increased by 5, 3, and 2-fold, respectively, in the B16F1 close to HUVEC (within 80 µm distance) compared to that far from HUVEC (over 80 µm distance) (Fig. 6). This 80 µm distance from HUVEC was the distance that increased invasive morphology of B16F1 cells in Fig. 5. Since IL-6 and MMP-9 are important for invasion [6]–[10], the increase of these gene expressions was comparable to the invasive cell behaviors that exist within 80 µm from HUVEC displayed in Fig. 5. These gene expression levels of cytokines related to tumor invasion, and the gene expression level of drug-efflux transporter related to drug resistance in melanoma, indicating that the proximity to vascular endothelial cell enhanced melanoma malignancy. Therefore, the melanoma spheroid arrays co-cultured with vascular endothelial network on the Matrigel base layer with embedded collagen type-I demonstrate the importance of vascular network in invasive cell behaviors in bioengineered tumor microenvironments.

## Discussion

Microengineering techniques for cellular analysis are gaining momentum as powerful tools to study cellular events for tissue-engineering and medical applications. In the field of cancer treatment, discovery of key factors affecting cell invasion and metastasis would be possible using *in vitro* 3D cell culture models. Recently, *in vitro* 3D cell culture systems that mimic tumor microenvironments have attracted much attention for deepening understanding and hastening the development of treatment, since these models contain the structural architecture necessary for studying cellular interactions. Such systems possess distinct advantages over conventional cell culture systems, including the ability to produce 3D architecture with controlled and repeatable spatial relationships between the cells in ECM. Therefore, applying tissue-engineering concepts and microengineering techniques in these systems would be expected to bridge the gap between two-dimensional studies and *in vivo* animal models [35].

In the present study, we have investigated the effect of a vascular formed endothelial cell network on the invasive capacity of melanoma in a biomimetic microenvironment using the *in vitro* 3D co-culture patterning model (Fig. 1). It was demonstrated that through interactions of tumor cells with vascular endothelial cells, genetic expression orchestrating tumor invasion was enhanced (Fig. 3, 4, 6A). Thus, a vascular endothelial network with cellular and ECM components plays a crucial role in regulating the process of tumor invasion. It can be deduced that traditional 2D cultures or even 3D monocultures without ECM or vascular endothelial cells give inaccurate representations of cancer invasion or genetic progression than that of the natural tumor microenvironment. In addition, it was clearly shown that the proximity to a vascular endothelial network accelerated the cancer invasive behavior and tumor-associated gene expression (Fig. 3, 5, 6B, 6C). It is known that numerous kinds of cytokines including TNF-α secreted by HUVEC [4] have regulated malignant capacity and drug resistance of cancer cells [3], [34], [36]–[38]. However, since most cancer cells also overproduce various kinds of proteinase that promote digestion of ECMs and cytokines in the cancer microenvironment [39]–[41], the local concentration of these cytokines secreted by HUVEC should be decreased according to the distance from the vascular network. Therefore, we considered that the malignant changes of invasive behavior and gene expression of B16F1 were caused by the difference in exposure amount of cytokines according to the distance from HUVEC. Additionally, since intercellular adhesion also regulates cell growth, motility, and angiogenesis via N-cadherin or PKC signals [32], [34], many researchers have been focused on adhesion-mediated malignancy within tumors to understand the mechanism of metastasis. In this research, MDR-1 expression in spheroids adhered to HUVEC was higher than that of non-adherent spheroids within 80 µm distance from HUVEC (Fig. S1), since MDR-1 expression has been stimulated via both paracrine signaling such as TNF-α and cell-to-cell adhesion [34], [37]. In contrast, there were no significant differences of IL-6 and MMP-9 expressions in the adhered spheroids to HUVEC were comparable to that in non-adherent spheroids within 80 µm distance from HUVEC (Fig. S1), since these expressions were stimulated by paracrine signaling such as TNF-α [3], [36]–[38]. Thus, applying genetic engineering techniques such as fluorescent protein linked with the adhesion molecule in these systems, the cellular micropatterning method can provide useful models to investigate paracrine signaling and intercellular adhesion with high resolution real time monitoring. Our micropatterning method could clearly detect the increases of IL-6 expressions in individual B16F1 melanoma spheroids in each distance from HUVEC (Fig. 6B, 6C), while the average expression of whole B16F1 cells co-cultured with HUVEC showed no significant increase with that without HUVEC (Fig. 6A). Our co-culture model enables the observation of local changes in cell morphology as well as their gene expressions and leads to understanding of the cancer microenvironment.

Tumor cell behavior is regulated by its intrinsic properties as well as by its microenvironment, which comprises resident endothelial cells, ECM, and fibroblasts [28]. The fibroblasts involved in primary tumor formation and invasion are referred to as cancer associated fibroblasts (CAFs), we had evaluated the morphological and genetic interactions of melanoma in co-culture patterning model with fibroblasts using pin-holder device in our previous paper [27], [28]. However, the elongation of the B16F1 melanoma spheroids was changed only slightly in co-culture array with line-patterned normal human dermal fibroblasts (NHDFs). Line patterning devise was newly fabricated (Figure S2A). The magnetically labeled NHDF was inoculated on a thin layer of Matrigel for the line patterning at seeding density of 3×10^5^ cells/dish (as same as the cell seeding density of HUVEC), and co-cultured with magnetically labeled B16F1 array at seeding density of 10 cells/spot (Fig. S2). Although we observed the active elongation and invasion of melanoma spheroids within an 80 µm distance from HUVEC, the B16F1 melanoma spheroids co-cultured with NHDF did not almost move. In addition, Cedric Gaggioli et al had reported that the squamous cell carcinoma (SCC) moved in groups and SCCs were always close to fibroblasts, appearing to move ‘along’ them [42]. The SCCs was activated the invasive capacity by the adhesion signaling such as integrin α3 and α5, indicating that cancer cells were passively activated the invasive capacity by CAFs. Therefore, the activation of the B16F1 invasiveness in our report suggested that HUVEC played a crucial role in cancer invasion than the surrounding fibroblast, and our co-culture model with HUVEC was suitable for the observation of cancer invasive capacities than other co-culture models using the fibroblasts.

Most conventional 3D invasion assays, such as microfluidic models or microwell platform analysis, have the complexity of building platforms, and those models have difficulty in cell manipulation for subsequent biological analysis. Therefore, many cancer and molecular biologists do not widely use 3D culture techniques for invasion assay and practical drug screening models [41]. On the other hand, our methodology is simple to construct, easy to handle and uses generally available cell culture dishes for cell patterning and culture. For analysis, manipulation of the target cell spheroids from the 3D spheroid array could be performed simply and directly using a micromanipulator. Thus, the 3D cell culture array has remarkable advantages for practical invasion model and several biological analysis of target cell. In addition, since 3D cell patterning can arbitrarily design spatial position of the target cells, it is effective to observe the various interactions of cancer cells with the co-existent somatic cells in the tumor environment. The spatial control of magnetically labeled HUVEC is also possible by magnetic patterning of HUVEC in network formation [26], which leads to the development of a vascular networking model in a larger spheroid. Also, the co-existence of stromal spheroids is possible by mixing with the collagen gel or laying the cellular sheet [28], which leads to further investigation of intercellular interactions, such as among cancer-vascular-stromal cells mimicking the progression of the tumor microenvironment. This *in vitro* 3D magnetic force-based cellular array technique is a functional tumor model that can be used in the future to elucidate the invasive capacity of tumor cells as well as their pharmacological responses.

## Conclusions

The 3D spheroid cell array was developed by magnetic cell patterning for evaluation of the effect of associated vascular endothelial cells on invasion of tumor cells. This model was suitable for visualization of the intravasation events in real-time and for precise measurement of cell-cell distance. The crucial effect on the invasive behavior of melanoma was investigated by spheroid cell manipulation in an *in vitro* 3D cell culture platform of a co-culture array, and accelerated spreading and tumor-associated gene expressions (IL-6, MDR-1, and MMP-9) were significantly observed in the proximity of a vascular-like endothelial cell network. This demonstrates the fact that the 3D cell spheroid array is a valuable biomimetic model allowing for intercellular and ECM interactions. Therefore, the *in vitro* 3D magnetic force-based cell patterning method is a highly applicable technique for analysis, diagnostics, and drug screening in a biomimetic microenvironment.

## Supporting Information

Figure S1 **Gene expressions in B16F1 spheroids that adhered or non-adhered to HUVEC.** The expression levels in each B16F1 spheroid placed that adhered and non-adhered (≤80 µm) to HUVEC was compared. Expression data was normalized to each gene expressions found in B16F1 monoculture using GAPDH as the reference gene.
(TIF)

Figure S2 **The length of B16F1 spheroids co-cultured with the line patterning of fibroblast.** (A) The pin-holder device for creating the line patterning of human fibroblast cell line NHDF with different spacing. The center-to-center distance of the pin-holder device was 1 mm to 7 mm, and the cells were arranged on pins according to magnetic force. (B) Schematic diagram for fabrication of the 3D cell culture array. The cell culture dish with a thin layer of Matrigel was placed on the pin-holder device with line patterning which is placed on the neodymium magnet. The NHDF, labeled with MCL and celltracker orange, was inoculated on a thin layer of Matrigel for the line patterning at seeding density of 3×10^5^ cells/dish, followed by 30 min incubation. The pin-holder device and the magnet were then removed from the culture dish. After 1-day culture, the cell culture dish was placed on the pin-holder device with array patterning which is placed on the neodymium magnet. The B16F1, labeled with MCL and celltracker green, were patterned on the line patterning of NHDF for 30 min at seeding density of 10 cells/spheroid (1.8×10^5^ cells/dish). The patterned cells were then embedded with collagen gel, the pin-holder device and the magnet were then removed from the culture dish. (C) Magnetically labeled B16F1 cells were arrayed at seeding density of 10 cells/spheroid over NHDF lines. Time-lapse images were taken for three plates on 0 h and after 24 h. White arrows highlight B16F1 cells that have elongated with the NHDF. Scale bar: 100 µm. (D) The length of B16F1 cell spheroids patterned in 10 cells/spheroid with 250 µm interval were image-analyzed by the green fluorescence after a 24 h culture with the line patterning of NHDF. The plot represents the length of each B16F1 spheroid. The solid and dotted lines show the average length and the average length ±3× SD of B16F1 cell spheroids in 3D cell monoculture array.
(TIF)
