# Supplementary figures and images for: Effect of Vascular Formed Endothelial Cell Network on the Invasive Capacity of Melanoma Using the In Vitro 3D Co-Culture Patterning Model
Source: PLoS One. 2014 Jul 24;9(7):e103502. doi: 10.1371/journal.pone.0103502 (PMC4110033; doi:10.1371/journal.pone.0103502)

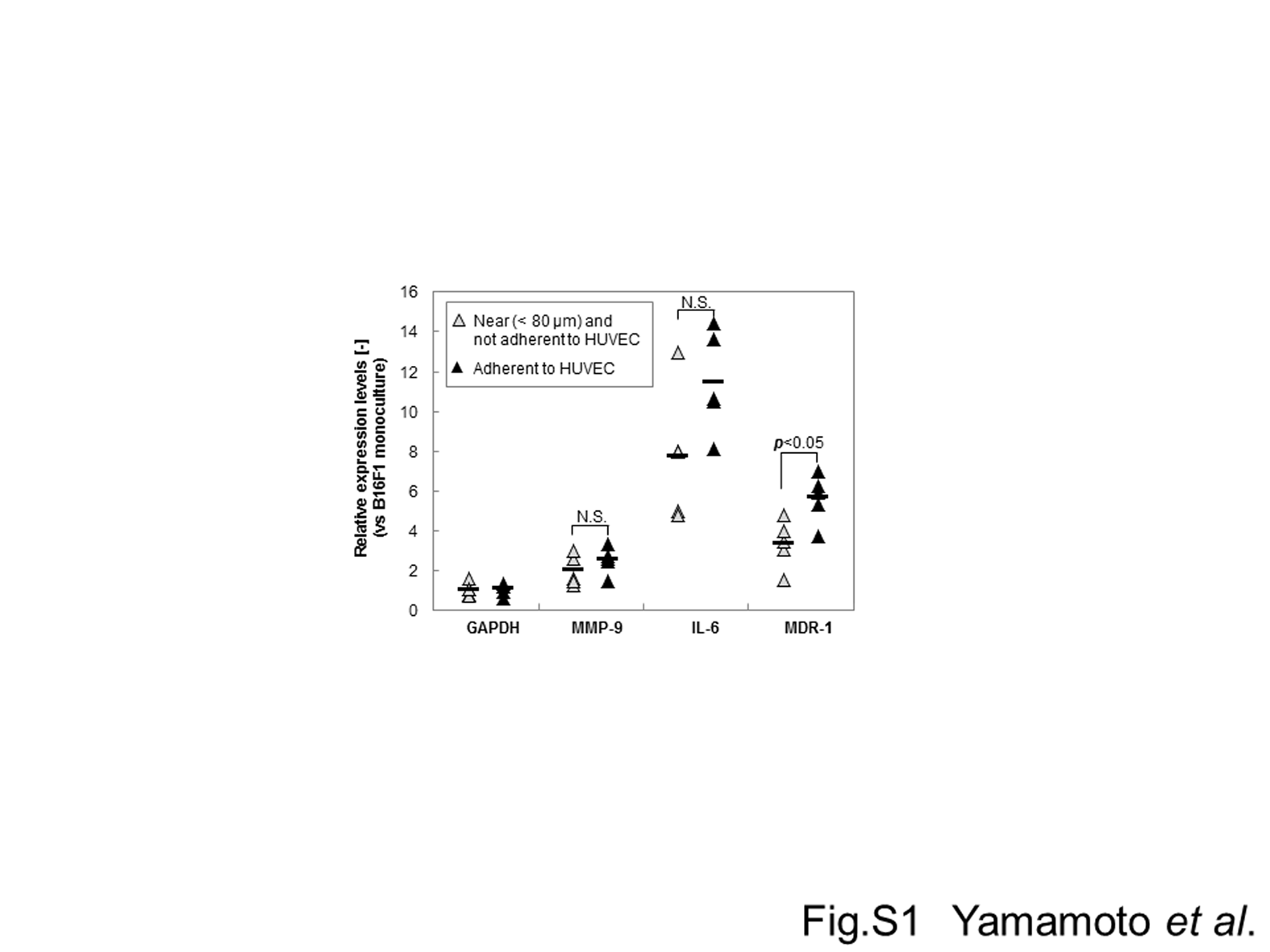

Supplement: Figure S1 — Gene expressions in B16F1 spheroids that adhered or non-adhered to HUVEC. The expression levels in each B16F1 spheroid placed that adhered and non-adhered (≤80 µm) to HUVEC was compared. Expression data was normalized to each gene expressions found in B16F1 monoculture using GAPDH as the reference gene. (TIF) [file pone.0103502.s001.tif]

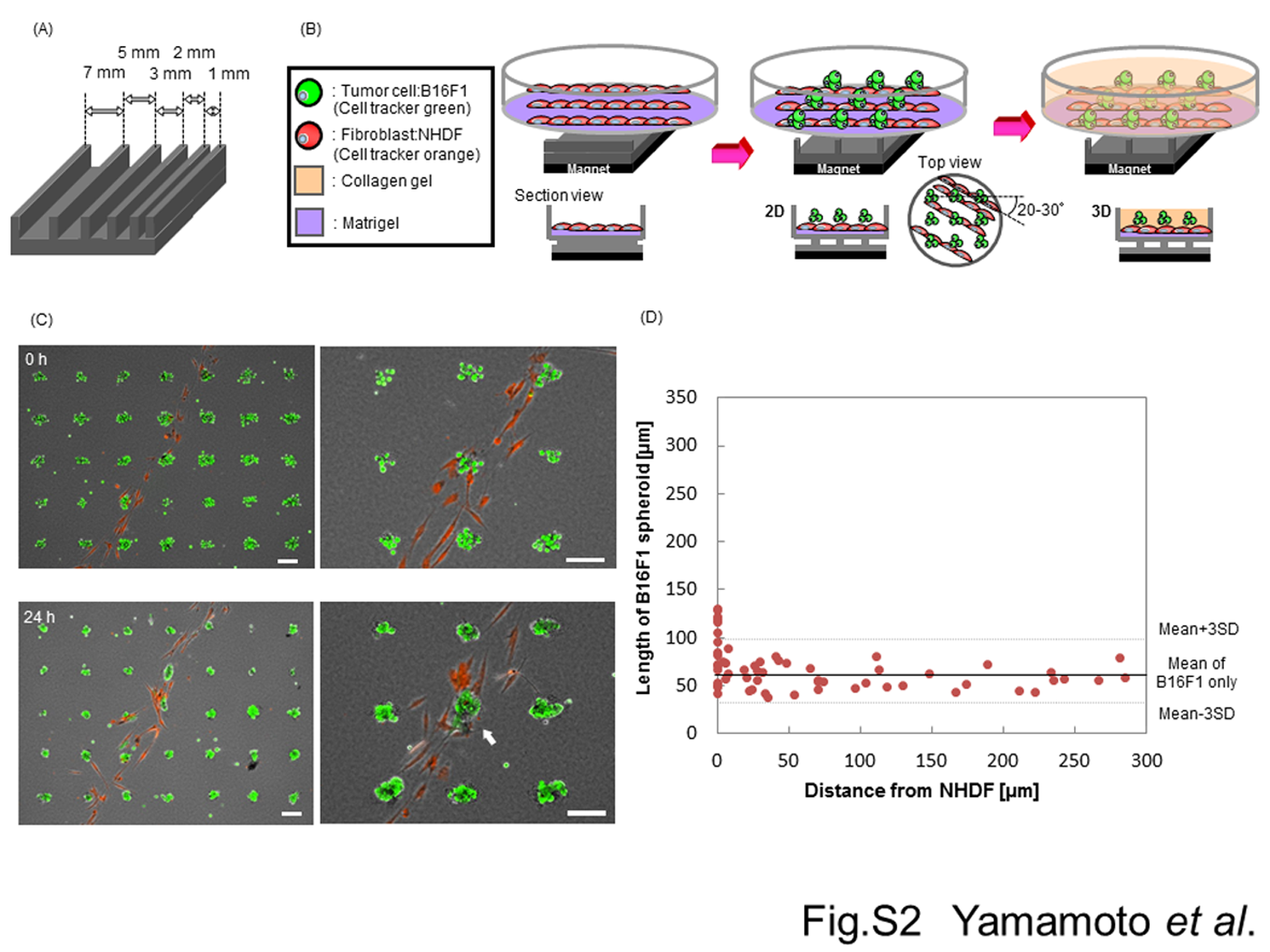

Supplement: Figure S2 — The length of B16F1 spheroids co-cultured with the line patterning of fibroblast. (A) The pin-holder device for creating the line patterning of human fibroblast cell line NHDF with different spacing. The center-to-center distance of the pin-holder device was 1 mm to 7 mm, and the cells were arranged on pins according to magnetic force. (B) Schematic diagram for fabrication of the 3D cell culture array. The cell culture dish with a thin layer of Matrigel was placed on the pin-holder device with line patterning which is placed on the neodymium magnet. The NHDF, labeled with MCL and celltracker orange, was inoculated on a thin layer of Matrigel for the line patterning at seeding density of 3×10^5^ cells/dish, followed by 30 min incubation. The pin-holder device and the magnet were then removed from the culture dish. After 1-day culture, the cell culture dish was placed on the pin-holder device with array patterning which is placed on the neodymium magnet. The B16F1, labeled with MCL and celltracker green, were patterned on the line patterning of NHDF for 30 min at seeding density of 10 cells/spheroid (1.8×10^5^ cells/dish). The patterned cells were then embedded with collagen gel, the pin-holder device and the magnet were then removed from the culture dish. (C) Magnetically labeled B16F1 cells were arrayed at seeding density of 10 cells/spheroid over NHDF lines. Time-lapse images were taken for three plates on 0 h and after 24 h. White arrows highlight B16F1 cells that have elongated with the NHDF. Scale bar: 100 µm. (D) The length of B16F1 cell spheroids patterned in 10 cells/spheroid with 250 µm interval were image-analyzed by the green fluorescence after a 24 h culture with the line patterning of NHDF. The plot represents the length of each B16F1 spheroid. The solid and dotted lines show the average length and the average length ±3× SD of B16F1 cell spheroids in 3D cell monoculture array. (TIF) [file pone.0103502.s002.tif]
